# A successful treatment of severe systemic lupus erythematosus caused by occult pulmonary infection-associated with hemophagocytic syndrome: A case report

**DOI:** 10.1097/MD.0000000000010595

**Published:** 2018-05-11

**Authors:** Weihong Shi, Mingyang Duan, Ligang Jie, Weifeng Sun

**Affiliations:** a Department of Chinese Medicine, Guangzhou General Hospital of Guangzhou Military Command; b Guangzhou University of Chinese Medicine, Guangzhou, China.

**Keywords:** hemophagocytic syndrome, hemophagocytosis, systemic lupus erythaematosus

## Abstract

**Rationale:**

A 27-year-old woman with a history of systemic lupus erythaematosus (SLE) developed hemophagocytic syndrome (HPS) secondary due to an unrecognized infection that led to severe SLE with a prolonged recovery.

**Patient concerns:**

The patient showed a high spiking fever and myalgia. Laboratory data revealed pancytopenia and immunological abnormalities. Pulse methylprednisone plus intravenous immunoglobulin (IVIG) failed to improve the clinical symptoms and laboratory data.

**Diagnoses:**

As activated macrophages with hemophagocytosis were confirmed in bone marrow histology, the patient was diagnosed as having reactive HPS.

**Interventions and outcomes:**

Her reactive HPS was successfully treated with intravenous antibiotics and was followed by oral prednisolone and hydroxychloroquine maintenance therapy.

**Lessons:**

In severe SLE, patients with persistent high fever, cytopenia, and elevated levels of serum ferritin and liver enzymes should be strongly suspected of reactive HPS, and aggressive examination, such as bone marrow biopsy, needs to be considered for early diagnosis and proper treatment.

## 1 Introduction

Systemic lupus erythaematosus (SLE) is an autoimmune disease characterized by widespread inflammatory involvement of the connective tissues. For this reason, many clinical manifestations have been found with various primary manifestations including musculoskeletal, cutaneous, constitutional, neurologic, and renal involvement, as well as lymphadenopathy and Raynaud's phenomenon. Hemophagocytic syndrome (HPS) is a clinicopathological entity characterized by the activation of macrophages and histiocytes with prominent hemophagocytosis in bone marrow and other reticuloendothelial systems.^[1]^ The hallmark of this syndrome is suggested as massive hypersecretion of proinflammatory cytokines induced by activated T-lymphocytes and macrophages that play an important role in the pathogenesis of HPS.^[2]^ There are 2 main forms of HPS: primary (familial) and secondary (reactive, acquired). Primary HPS denotes the presence of an underlying genetic disorder and is observed mostly in infants. Reactive HPS may develop as a rare but potentially fatal complication of several disorders, including various infections, autoimmune systemic diseases, malignancies, and administration of certain drugs.^[3–5]^ The exclusion of concurrent malignancies, and autoimmune disorders as the cause of HPS is very important for the establishment of the right therapeutic strategy. In severe SLE, infections can be difficult to distinguish from lupus flares because of similar clinical presentations. Herein, we report the case of a patient with intractable HPS who had undergone nonselective immunosuppressive therapy with IVIG and intravenous methylprednisolone (mPSL) pulse therapy twice, and she was the only one who improved after antibiotics were administered.

## 2 Case presentation

The study was approved by the Institutional Review Board for the Protection of Human Subjects of Guangzhou General Hospital of Guangzhou Military Region and adhered to the tenets of the Declaration of Helsinki. Informed consent was obtained from the patient

A 27-year-old Chinese woman with SLE was admitted to our hospital in October 2016 because of a high fever and myalgia. She had been diagnosed with SLE 6 years before this admission, based on findings such as arthritis, proteinuria, and livedo reticularis, as well as positivity for antinuclear antibody, anti-RNP antibody, anti-SSA/SSB antibody, and anti-SM antibody. She was treated with oral prednisolone, mycophenolate, thunder god vine, and hydroxychloroquine daily, and as a result, her symptoms were much improved. Her disease had been well controlled for 2 years and the dose of oral prednisolone was gradually reduced and maintained at 10 mg and 400 mg hydroxychloroquine daily.

Twenty days before the present admission, she complained about prolonged fever without a specific pattern. Followed by treatment at a local hospital, the fever was not reduced after 3-day steroid pulse therapy with 500 mg/day methylprednisolone; soon afterward, 20 g/day of intravenous immunoglobulin (IVIG) was administered for 3 days, but this treatment was ineffective. She was given broad-spectrum antibiotics and antifungal agents for 7 days. However, no apparent improvement was observed in her condition. When she visited our hospital, she was in poor condition.

Upon hospital admission, she appeared rather well and in no distress except for a body temperature of 37.7°C. Her blood pressure, pulse rate, and respiratory rate were normal. There were no palpable lymph nodes. Examination of heart and lungs showed no abnormal findings. Her abdomen was soft, not tender, and not distended; her liver and spleen were not palpable. She had no arthritis in the upper or lower extremities. No oedema was observed, despite findings of hypoalbuminemia. Neurological findings were also normal.

Complete blood cell counts, which were performed after admission, showed leukopenia (white blood cell count at 1.22 × 109/L), lymphopenia (lymphocyte count at 0.68 × 109/L), and normocytic normochromic anaemia (hemoglobin at 10.3 g/dL and red blood cell count at 3.78 × 1012/L). The results of blood chemistry were AST 371 IU/L (reference range, 0–40 IU/L), ALT 121 IU/L (reference range, 0–50 IU/L), lactate dehydrogenase 1691 U/L (reference range, 109–245 U/L), C3 0.23 g/dL (reference range, 0.9–1.8 g/dL), and C4 0.123 g/dL (reference range, 0.1–0.4 mg/dL). The ESR was 41 mm/h, and the CRP level was also elevated to 17.9 mg/dL (reference range, 0–6 mg/dL). Serum ferritin was markedly elevated to>2000 ng/mL (reference range, 4.63–204 ng/mL). Activated partial thromboplastin time 56.7 sec; fibrinogen 1.8 g/l, thrombin time (TT) 27.9 sec; urinalyses showed positive protein, negative occult blood, and urinary excretion of protein accounted for 0.98 g/day. All investigations resulted from the present hospital admission, including tuberculin test, sputum smear for tuberculosis, and infection related viral and bacterial cultures showed negative results (Table 1).

**Table 1:**
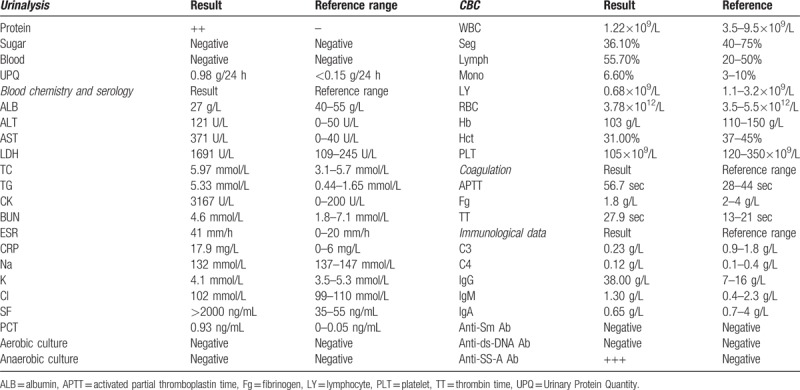
Laboratory data upon hospital admission.

**Figure 1:**
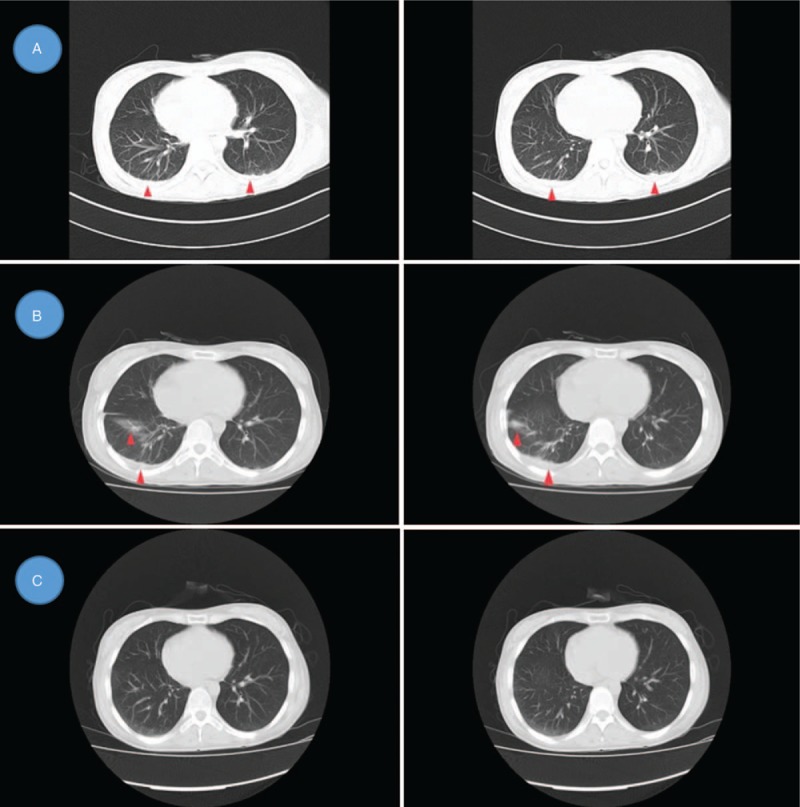
(A) Lung CT scan detected the bilateral pleural effusion. (B) On day 23, lung CT was reviewed: the right lower lobe had new inflammation, the right pleural effusion increased more than before. (C) The lung CT was reviewed before discharge and the lung infection was eliminated. CT, computed tomography.

**Figure 2:**
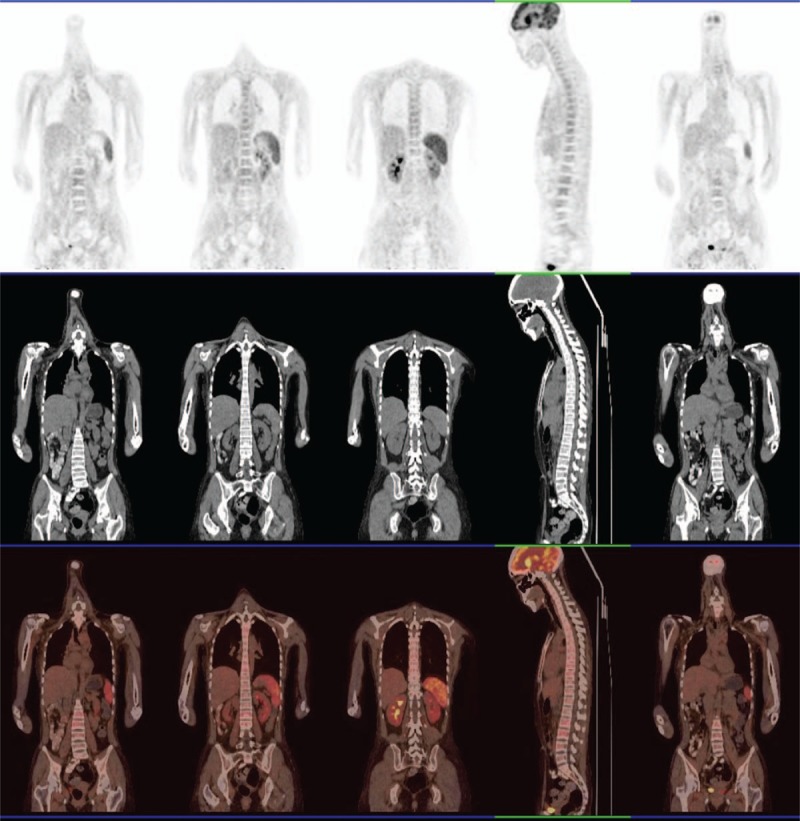
Malignant tumours and abscesses not found by PET/CT scan.

**Figure 3:**
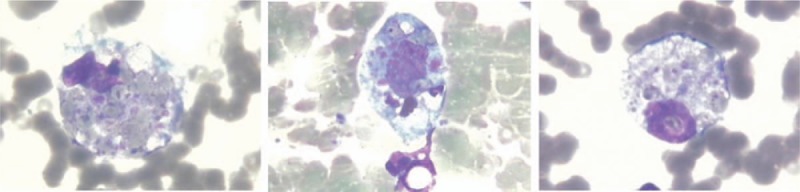
Bone marrow aspiration showed hemophagocytic syndrome.

**Figure 4:**
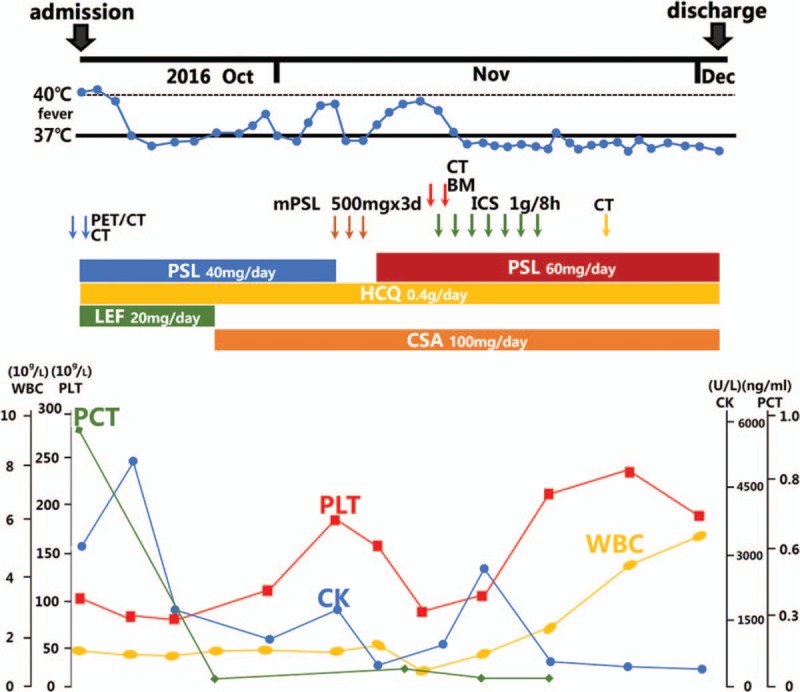
Clinical course of the patient.

Ultrasound cardiography did not detect pericardial effusion. Lung CT scan detected bilateral pleural effusion (Fig. 1A). Positron emission tomography/computed tomography (PET/CT) scan was performed to search for malignant tumours and abscesses, but no abnormalities were found other than mild bilateral pleural effusion, mild splenomegaly and a systemic increase in lymph nodes (Fig. 2). SLE had a high activity index (SLEDAI was 13), a lupus flare was suspected, pulse methylprednisolone (500 mg/day for 3 days) was administered, and high-dose prednisolone therapy was continued thereafter (60 mg/day). However, despite high-dose corticosteroid treatment, her fever, cytopenia, and elevated lactate dehydrogenase continued. Multiple cultures of blood and bone marrow for other fungi, bacteria and viruses were negative. Therefore, we performed biopsy of the bone marrow to identify its pathology. The bone marrow smear showed hemophagocytosis: macrophages engulfing lymphocytes, erythrocytes and platelets (Fig. 3). Hemophagocytic syndrome was diagnosed. On day 23, the lung CT was reviewed: the right lower lobe had new inflammation, and the right pleural effusion increased more than before (Fig. 1B). After an extensive study, the causative underlying disease was uncertain, considering the ineffective pulse methylprednisolone therapy, it was not the SLE itself. The patient was diagnosed with reactive HPS related to an infection. Though no serum evidence of infection has been found in these patients, lung CT showed a new infection, which strongly indicated that HPS could be triggered by infection. She started intravenous pulse antibiotic agents (Imipenem and Cilastatin Sodium) for 7 days (1 g/8 h), with maintenance of high-dose prednisolone. The high fever disappeared the day following the end of Imipenem and Cilastatin Sodium therapy, and myalgia also disappeared after about one week. Blood tests performed after a week showed resolved pancytopenia, with the exception of anaemia (hemoglobin at 10.7 g/dL). The serum levels of procalcitonin became normal. Proteinuria decreased to 0.26 g/day. Serum complement C3 and C4 levels were both elevated (C3 level: 0.96 g/L, C4 level: 0.31 g/L), with a high level of total cholesterol (10.27 mmol/L). The ESR was 15 mm/h, and CRP level was under 3.16 mg/dL. HPS did not recur and the prednisolone dose was slowly reduced, subsequently. The lung CT was reviewed before discharge and the lung infection was eliminated (Fig. 1C). The patient was discharged in early December and all of the laboratory findings returned to normal values (Fig. 4). The SLEDAI value estimated before discharge was 0, indicating that the disease could be considered to be in remission. The patient was followed-up for half a year after discharge from the hospital. She is currently being treated with 20 mg of prednisolone, and 200 mg hydroxychloroquine per day. Blood cell count and the ferritin levels were within the normal ranges, and there was no relapse of SLE or HPS.

## 3 Discussion

HPS, pulmonary infection, interstitial pneumonia, and polymyositis are life-threatening, fortunately rare, complications of SLE. Severe autoimmune disorders cause substantial morbidity and mortality, however. HPS is a rare but potentially life-threatening disease. Cardinal symptoms of HPS are prolonged high fever, lymph node swelling, and hepatosplenomegaly. Characteristic laboratory findings include high triglycerides, ferritin, transaminases, and decreased fibrinogen. Upon histology, active macrophages with ingestion of cellular blood components and their precursor cells are the typical findings. The hemophagocytosis can be observed, particularly, in the bone marrow, lymph nodes, liver, and spleen.^[6,7]^ It is not sufficient to rely solely on bone marrow histopathological findings as hemophagocytosis may not appear in the bone marrow biopsy in the early phases of HPS.^[8]^ If this feature is absent in the initial biopsy specimen, biopsy should be repeated.^[9]^ Diagnosis of HPS relies on clinical, laboratory, and histopathological findings. HPS occurring in the course of systemic disease is an infrequent but important clinical entity in terms of patient prognosis. HPS linked exclusively to acute SLE is rare. Currently, there are no pathognomonic clinical or laboratory parameters for the diagnosis of reactive HPS, and a treatment strategy for reactive HPS in SLE patients has not been established. HPS and SLE share several clinical features and similar pathogenic abnormalities. It is difficult to early identify HPS in patients with SLE, but it is important because it can lead clinicians to initiate therapy that directly affects the morbidity and mortality of patients. As SLE patients with HPS have higher disease severity and are complicated with severe conditions, such as infection with virus or bacteria, and with multiple organ damage, it is important that physicians immediately search for the underlying disease and a possible infectious trigger. However, the clinical symptoms include polymyositis and panhaematopenia, and the most likely cause was thought to be an SLE flare; thus, the underlying disease caused by reactive HPS was neglected. This patient underwent mPSL pulse therapy twice and was ineffective. Because the patient serum PCT, CRP, and ferritin levels seemed to be markedly higher than that usually found in SLE patients, we suspected the presence of an infectious disease of undetected origin. Severe pulmonary involvement, including pleuritis and hydrothorax in patients, is associated with lung infection. According to fever, hyperferritinaemia, liver dysfunction, hyperlipemia, and pancytopenia symptoms, SLE complicating HPS was suspected. In addition, soon afterward, hemophagocytic macrophages were confirmed in bone marrow biopsy, besides, PET/CT scan excluded malignant tumours, such as lymphoma. We hypothesized that the lung infection was the trigger for HPS in our case and led to a severe flare of the underlying SLE. It led us to initiate therapy with infectious pneumonia, and she was also treated with senior antibiotics and a medium dose of oral corticosteroids. Following this combined therapy, the serum ferritin and triglyceride levels were reduced in association with improvements in her clinical condition. Pulse methylprednisolone and immunosuppressive agents may cause a weakened immune state, thus increasing the possibility of the infection. As evidence of infection had been found in patients, it strongly indicated lupus activity could be triggered by HPS. However, if an opportunistic infection occurs while the patient is receiving immunosuppressive therapy, the dose of the immunosuppressive drugs needs to be reduced and antibiotic therapy should be instituted. Currently, there are no pathognomonic clinical or laboratory parameters for the diagnosis of HPS. Activated macrophages may release ferritin, and the elevated serum ferritin levels reflect the activation of macrophages to a certain extent.^[10]^ The presence of serum ferritin is the most important indicator of HPS.

When unexplained high spiking fever, high serum ferritin, liver dysfunction, and cytopenia in 2 or more cell lineages progress during the course of an autoimmune disease, the physician should be aware of the possibility of HPS. HPS is an uncommon manifestation in SLE, and its occurrence can be associated with events that alter lupus disease activity or infections. Physicians should recognize the clinical entity, Improve the and aggressive examination to provide accurate infection diagnosis, sufficient supportive intravenous antibiotics care, which are critical in improving patients’ prognosis.

## Author contributions

**Software:** Mingyang Duan, Ligang Jie.

**Writing – original draft:** Weihong Shi.

**Writing – review & editing:** Weihong Shi, Weifeng Sun.

## References

[1] Imashuku S Differential diagnosis of hemophagocytic syndrome: underlying disorders and selection of the most effective treatment. Int J Hematol 1997;66:135. 9277044 10.1016/s0925-5710(97)00584-7

[2] Janka GE Familial and acquired hemophagocytic lymphohistiocytosis. Eur J Pediatr 2007;166:95–109. 17151879 10.1007/s00431-006-0258-1

[3] Grom AA Macrophage activation syndrome and reactive hemophagocytic lymphohistiocytosis: the same entities. Curr Opin Rheumatol 2003;15:587–90. 12960485 10.1097/00002281-200309000-00011

[4] Castillo L Carcillo J Secondary hemophagocytic lymphohistiocytosis and severe sepsis/systemic inflammatory response syndrome/multiorgan dysfunction, syndrome/macrophage activation syndrome share common, intermediate phenotypes on a spectrum of inflammation. Pediatr Critic Care Med 2009;10:387–92. 10.1097/PCC.0b013e3181a1ae08 19325510

[5] Canna SW Behrens EM Not all hemophagocytes are created equally: appreciating the heterogeneity of the hemophagocytic syndromes. Curr Opin Rheumatol 2012;24:113–8. 22089101 10.1097/BOR.0b013e32834dd37e PMC3285509

[6] Fisman DN Hemophagocytic syndromes and infection—statistical data included. Emerging Infect Dis 1999;6:601. 10.3201/eid0606.000608 PMC2640913 11076718

[7] Janka GE Lehmberg K Hemophagocytic syndromes—an update. Blood Rev 2014;28:135. 24792320 10.1016/j.blre.2014.03.002

[8] Gupta A Tyrrell P Valani R The role of the initial bone marrow aspirate in the diagnosis of hemophagocytic lymphohistiocytosis. Pediatr Blood Cancer 2008;51:402. 18523990 10.1002/pbc.21564

[9] Rouphael NG Talati NJ Vaughan C Infections associated with hemophagocytic syndrome. Lancet Infect Dis 2008;7:814–22. 10.1016/S1473-3099(07)70290-6 PMC7185531 18045564

[10] Lambotte O Cacoub P Costedoat N High ferritin and low glycosylated ferritin may also be a marker of excessive macrophage activation. J Rheumatol 2003;30:1027–8. 12734900

